# Changes of CD103-expressing pulmonary CD4^+^ and CD8^+^ T cells in *S. japonicum* infected C57BL/6 mice

**DOI:** 10.1186/s12879-019-4633-8

**Published:** 2019-11-27

**Authors:** Yi Zhao, Quan Yang, Chenxi Jin, Yuanfa Feng, Shihao Xie, Hongyan Xie, Yanwei Qi, Huaina Qiu, Hongyuan Chen, Ailin Tao, Jianbing Mu, Wenjuan Qin, Jun Huang

**Affiliations:** 1 Sino-French Hoffmann Institute, Guangdong Provincial Key Laboratory of Allergy & Clinical Immunology, The State Key Laboratory of Respiratory Disease, The Second Affiliated Hospital of Guangzhou Medical University, Guangzhou, 510260 China; 2 Guangdong Pharmaceutical University, Guangzhou, 510006 China; 3 Laboratory of Malaria and Vector Research, National Institute of Allergy and Infectious Diseases, National Institutes of Health, Bethesda, MD USA; 4 Department of Radiation Oncology, Zhongshan Hospital Xiamen University, Xiamen, 361004 China

**Keywords:** *Schistosoma japonicum*, Lung, CD103^+^ T cells, Phonotype, Cytokines

## Abstract

**Background:**

Recent studies have shown that CD103 is an important marker for tissue-resident memory T cells (TRM) which plays an important role in anti-infection. However, the role of CD103^+^ TRM was not elucidated in the progress of *S. japonicum* infection induced disease.

**Methods:**

6–8 weeks old C57BL/6 mice were infected by *S. japonicum*. Mice were sacrificed and the lungs were removed 5–6 weeks after infection. Immunofluorescent staining and Q-PCR were performed to identify the expression of CD103 molecule. Single cellular populations were made, percentages of CD103 on both CD4^+^ and CD8^+^ T lymphocytes were dynamical observed by flow cytometry (FCM). Moreover, the expression of memory T cells related molecules CD69 and CD62L, T cell function associated molecules CD107a, IFN-γ, IL-4, IL-9, and IL-10 were compared between CD103^+^ CD4^+^ and CD8^+^ T cells by FCM.

**Results:**

CD103^+^ cells were emerged in the lung of both naive and *S. japonicum* infected mice. Both the percentage and the absolute numbers of pulmonary CD4^+^ and CD8^+^ cells were increased after *S. japonicum* infection (*P* < 0.05). The percentage of CD103^+^ cells in CD8^+^ T cells decreased significantly at the early stage of *S. japonicum* infection (*P* < 0.05). Increased CD69, decreased CD62L and CD107a expressions were detected on both CD4^+^ and CD8^+^ CD103^+^ T cells in the lungs of infected mice (*P* < 0.05). Compared to CD8^+^ CD103^+^ T cells, CD4^+^ CD103^+^ T cells from infected mice expressed higher level of CD69 and lower level CD62L molecules (*P* < 0.05). Moreover, higher percentage of IL-4^+^, IL-9^+^ and IL-10^+^ cells on CD4^+^ CD103^+^ pulmonary T cells was found in infected mice (*P* < 0.05). Significantly increased IL-4 and IL-9, and decreased IFN-γ expressing cells were detected in CD8^+^CD103^+^ cells of infected mice (*P* < 0.05).

**Conclusions:**

CD103-expressing pulmonary CD4^+^ and CD8^+^ T cells play important roles in mediating *S. japonicum* infection induced granulomatous inflammation in the lung.

## Background

Schistosomiasis is a chronic parasitic disease caused by the parasite *S. japonicum*. It is a major health risk for more than 50 million people [1]. About 3–4 weeks after *S. japonicum* infection, the eggs laid by adult worm mainly began to deposit in the liver leading to the formation of liver granuloma and fibrosis which was the typic pathological change [2]. Additionally, eggs could deposit in the lung and wall of intestinal tract of animals, and induced related diseases [3–5]. At 5–6 weeks after infection, obviously egg granulomatous inflammation could be detected in the liver and lung [6, 7].

Many kinds of immune cells were involved in the course of *S. japonicum* infection [8–10]. CD4^+^ T cells were reported to be the main population of lymphocytes mediating *S. japonicum* infection induced immune response in C57BL/6 mouse model, which could secrete many kinds of cytokines, including: IFN-γ, IL-4, IL-9, IL-10, and so on [11, 12]. Granzyme, tumor necrosis factor and perforin were the main cytotoxic factors produced by CD8^+^ T cells. Membrane expression of CD107a constitutes a marker of immune cell activation and cytotoxic degranulation [13]. Recently, many kinds of cytokines secreting by CD4^+^ T cells was found produced by CD8^+^ cells [14]. And CD8^+^ T cell was reported to involve in the progress of *S. japonicum* infection [15].

Memory T cells (Tm) is a small population of antigen specific T cell living in the lymph organs, which can response quickly and effectively to the re-encounter pathogens. According to the expression of CCR7 and CD62L, memory T cells could divide into central memory T cells and effective memory T cells [16]. Recently, a subpopulation of memory T cell that resides in peripheral tissues has been defined as tissue-resident memory T (TRM) cells, which could provide a first line of defense against infection at mucosal surfaces, responding rapidly without a need for recruitment of T cells from the circulation [17]. TRM cell was not involved in systemic circulation, but long-term settlement in specific tissues [18–20]. It was reported that liver resident memory CD8^+^ T cells form a front line defense against malaria liver stage infection [21]. Moreover, antigen-specific CD4 TRM cell induced by *Bordetella pertussis* infection played a critical role in adaptive immunity against re-infection [22].

CD103 belongs to the integrin family and is the E chain of integrin αEβ7 [23]. With the β7-binding integrin chain, CD103 is the E cadherin ligand that expressed on the barrier tissue on epithelial cells, intraepithelial lymphocyte T cells, regulatory T cells, dendritic cells, and mast cells, etc. [24–26]. CD103 expressing cells could distribute in the intestinal mucosa, lung, vagina, skin, kidney, lymph nodes and other tissues [27, 28]. Recent studies have shown that CD103 is also an important marker of tissue-resident memory T cells (TRM) [29]. CD69 is a classic marker for T cells early activation, which linked to TCR signal initiation in the past [30]. In addition, CD69 was served as the main marker for TRM cells, which could help TRM cells located in the tissues by inhabiting the activation of sphingosine-1-phosphate receptor 1 (S1PR1) [31]. According to the expression of CD103, CD69^+^ TRM could be divide two populations: CD69^+^CD103^+^ TRM cells and CD69^+^CD103^−^ TRM cells [17]. Integrin alpha 1, also known as VLA-1 (CD49a) could promote tissue retention and survival through binding to collagenase type IV, which is dispensable for TRM formation in the lung [32]. Programmed death-1 (PD-1) serves to limit the pathogenic capacity of exhausted-like TRM cells, blockade of PD-1 could reinforce the effect of a multiepitope vaccine, in boosting the frequency of HSV-1 specific CD8^+^ TRM cells and reducing disease severity [33]. Killer cell lectin-like receptor G1 (KLRG1) expressing cells receiving intermediate amounts of activating and inflammatory signals, differentiated into all memory T cell linages, including peripheral memory cells and TRM cells [34]. Activation molecule class I restricted T-cell associated molecule (CRTAM) expressing CD4^+^ and CD8^+^ TRM cells, which could traffic to mucosal tissues and inflammatory sites, were found localized in vaginal mucocutaneous (VM) tissues [35]. In addition, the molecule CD101 seems to be a strong co-stimulatory molecule for T cells, which has restricted expression predominantly on mucosal T lymphocytes, could enhance the activation of CD 103^+^TRM cells [36].

In this study, to characterize pulmonary CD4^+^ and CD8^+^ CD103^+^ cells in the progress of *S. japonicum* infection, C57BL/6 mice were infected by *S. japonicum*. The content and location of CD103 molecule was detected in the lung by Immuno-fluorescent staining and Q-PCR. The percentages of CD103 expressing both CD4^+^ and CD8^+^ T lymphocytes were dynamical observed by FCM. The expression of Tm cell related molecules CD69 and CD62L, T cell function associated molecules CD107a, IFN-γ, IL-4, IL-9, and IL-10 on CD4^+^ and CD8^+^ CD103^+^ cells were compared between naive and infected mouse by FCM.

## Methods

## Ethical statement

All protocols for animal use were approved to be appropriate and humane by the institutional animal care and use committee of Guangzhou Medical University (2012–11). All of the experiments were performed according to the guidelines of the Institutional Animal Committee of Guangzhou Medicine University. Every effort was made to minimize suffering.

### Experimental animals and infection

A total of eighty female C57BL/6mice, aged 6–8 weeks, 20-25 g, was purchased from Laboratory Animal Center of Sun Yat-sen University (Guangzhou, China) and maintained in a specific pathogen-free microenvironment at the Laboratory Animal Center, Guangzhou Medical University. The animals were maintained under the controlled environmental conditions of humidity, temperature and a light–dark cycle, with commercial balanced mouse chow and water provided ad libitum. *Oncomelania hupensis* infected snails were purchased from National institute of parasitic diseases Chinese Center for Disease Control And Prevention (Shanghai, China). Animals were acclimatized for at least 5 days before the start of any experimental procedure. Group sizes of 5–6 mice were used as indicated throughout the manuscript.

Mice were random divided into infected groups and naive control groups. The mouse in infected group was percutaneous infected with 40 ± 5 *S. japonicum* cercariae that were obtained from *Oncomelania hupensis* infected snails. To dynamical observe the percentages of CD103 expressing CD4^+^ and CD8^+^ T lymphocytes, mice were sacrificed weekly after infection from week 1 to week 7. To detect the phenotypic and functional character of CD4^+^ and CD8^+^ TRM cells, mice were sacrificed 5–6 weeks after infection.

### Antibodies

FITC conjugated anti-mouse CD3 (145-2C11), PerCPCy5.5 conjugated anti-mouse CD4 (RM4–5), PE conjugated anti-mouse CD103 (2E7), APC-cy7 conjugated anti-mouse CD8 (53–6.7), Alexa Fluor 647 conjugated anti-mouse CD103 (2E7), APC conjugated anti-mouse IL-4 (8D4–8), Alexa Fluor 647 conjugated anti-mouse IL-9 (MH9A3), APC conjugated anti-mouse IL-10 (JES5-16E3), and isotype-matched control monoclonal antibodies were purchased from BD (San Diego, CA, USA). BV510 conjugated anti-mouse CD45 (Antibody (30-F11), BV421 conjugated anti-mouse CD69 (H1.2F3), APC conjugated anti-mouse CD62L (MEL-14), APC conjugated anti-mouse CD107a (1D4B), FITC conjugated anti-mouse IFN-γ (XMG1.2) and their corresponding isotype controls were obtained from Bio-Legend (San Diego, CA, USA).

### Lymphocyte isolation

The isolated lung tissues were cut into small pieces and incubated in 5 mL digestion buffer (collagenase IV/DNase I mix, Invitrogen Corporation) at 37 °C for 30 min. The digested tissue fragments were pressed through the 200-mesh cell strainer and suspended in Hank’s balanced salt solution (HBSS). Lymphocytes were isolated using mouse lymphocyte separation medium (Dakewe Biotech) density gradient centrifugation. Lymphocytes in spleen were isolated using blood cell lysis buffer (Dakewe Biotech). The cells were washed twice (HBSS) by centrifugation (4 °C, 600 g, 5 min), and re-suspended at 2 × 10^6^ cells/ml in complete RPMI 1640 medium supplemented with 10% heat-inactivated fetal bovine serum (FBS), 100 U/ml penicillin, 100 μg/ml streptomycin, 2 mM glutamine, and 50 μM 2-mercaptoethanol.

### Histology studies

5–6 weeks after infection, the mice were sacrificed by cervical dislocation in laboratory. Lungs were removed from naive and infected mice, perfused three times with 0.01 M phosphate-buffered saline (pH = 7.4), fixed in 10% formalin, embedded in paraffin, and sectioned. The sections were then examined by light microscopy under 100× and 200× magnification after standard hematoxylin-eosin (H&E) staining for visualization of cellular changes under microscope (Leica SP8).

5–6 weeks after infection, lungs were removed from naive and infected mice, and frozen in optimal temperature compound (OCT) solution. The frozen lung tissues were sectioned. The frozen tissue sections were re-warmed at room temperature for 1 h, and washed with phosphate buffered saline (PBS) three times for 10 min/time to remove OCT. The sections were blocked with closed liquid (10% calf serum) and incubated overnight at 4 °C. Then, stain with CD103-Alexa Fluor 647 mAb (1:50 dilution) and incubate overnight (4 °C, in the dark). The sections were washed with PBS solution for three times. Sections were stained with DAPI in the dark for 10 min. Finally, sections were mounted in tablet, all prepared sections were observed in a dark room with laser scanning confocal microscopy (Leica SP8) .

### RNA preparation for real time PCR

Lymphocytes were performed following previously described procedures. Total RNA was isolated from the lung cells of infected and naive mice using Trizol Reagent (Invitrogen Life Technologies, Carlsbad, CA, USA) following the manufacturer’s instructions. The cDNA was synthesized, and mRNA expression was determined with a PrimeScript RT-PCR Kit (Takara, Tokyo, Japan) according to the manufacturer’s instructions. The CD103 primers were synthesized from Invitrogen (Shanghai, China) as follows: for 5′-GGG TCC TAC TTT GGC TCT GT-3′ (forward) and 5′-GTG TGT GTG CCA AGG AGA AG-3′ (reverse). The β-actin primers were synthesized from Invitrogen (Shanghai, China) as follows: 5′-CCG TAA AGA CCT CTA TGC CAA C-3′ (forward) and 5′-GGG TGT AAA ACG CAG CTC AGT A − 3′ (reverse). Real-time PCR amplification was performed using the CFX96 Touch fluorescent quantitative PCR (Bio-Rad, Hercules, CA). Amplification of β-actin was used as an internal control.

### Cell surface staining

The isolated pulmonary cells were washed twice with PBS and blocked in PBS buffer containing 1% BSA for 30 min. Cells were then stained for 30 min at 4 °C in the dark with conjugated antibodies specific for the cell surface antigens CD3, CD4, CD8, CD103, CD69, CD62L, and CD107a. The expression phenotypes of the antibody-labeled lymphocytes (1–2 × 10^6^ cells per run) were analyzed using a flow cytometer (Beckman CytoFLEX), and the results were analyzed using CytExpert 1.1 software (Beckman Coulter, Inc.). Isotype-matched controls for cytokines were included in each staining protocol.

### Intracellular cytokine staining

Single lymphocyte suspensions were isolated from the lungs of control and *S. japonicum*-infected mice, and the cell concentration was adjusted to 1.5 × 10^6^/ml. Cells were then stimulated with phorbol 12-myristate 13-acetate (PMA) (20 ng/ml, Sigma) and ionomycin (1 μg/ml, Sigma) for 5 h (37 °C, 5% CO_2_). Brefeldin A (BFA, 10 μg/ml, Sigma) was added during the last 4 h of incubation. Cells were washed twice in PBS and then stained for 30 min at 4 °C in the dark with conjugated antibodies specific for the cell surface antigens CD45, CD3, CD4, CD8, CD103 and CD69. Cells were fixed with 4% paraformaldehyde and permeabilized overnight at 4 °C in PBS buffer containing 0.1% saponin (Sigma), 1% BSA, and 0.05% NaN_3_.

Next, cells were stained with conjugated antibodies that were specific for cytokines IL-4, IFN-γ, IL-9, and IL-10. The expression phenotypes of the antibody labeled lymphocytes (2 × 10^6^ cells per run) were analyzed using a flow cytometer (Beckman CytoFLEX), and the results were analyzed using CytExpert 1.1 software (Beckman Coulter, Inc.). Isotype-matched controls for cytokines were included in each staining protocol.

### Expression and analysis of the results

The numerical data shown in the text and graphs represent percentages of the total number of cells identified according to the defined parameters. The data were analyzed with SPSS 13.0. A shapiro-Wilk’s test (*p* > 0.05) (Shapiro & Wilk, 1965) and a visual inspection of their histograms and normal Q-Q plots showed that the exam scores were approximately normally distributed for all data sets. In our study, Tukey’s honestly significant difference (HSD) post hoc tests are run to confirm where the differences occurred between groups. Means ± SD was reported, *P* < 0.05 was considered to be statistically significant. All experiments were performed independently a minimum of three times, using a minimum of three animals in each experimental group.

## Results

### Expression of CD103 molecule in the lungs of *S. japonicum*-infected mice

**Fig. 1:**
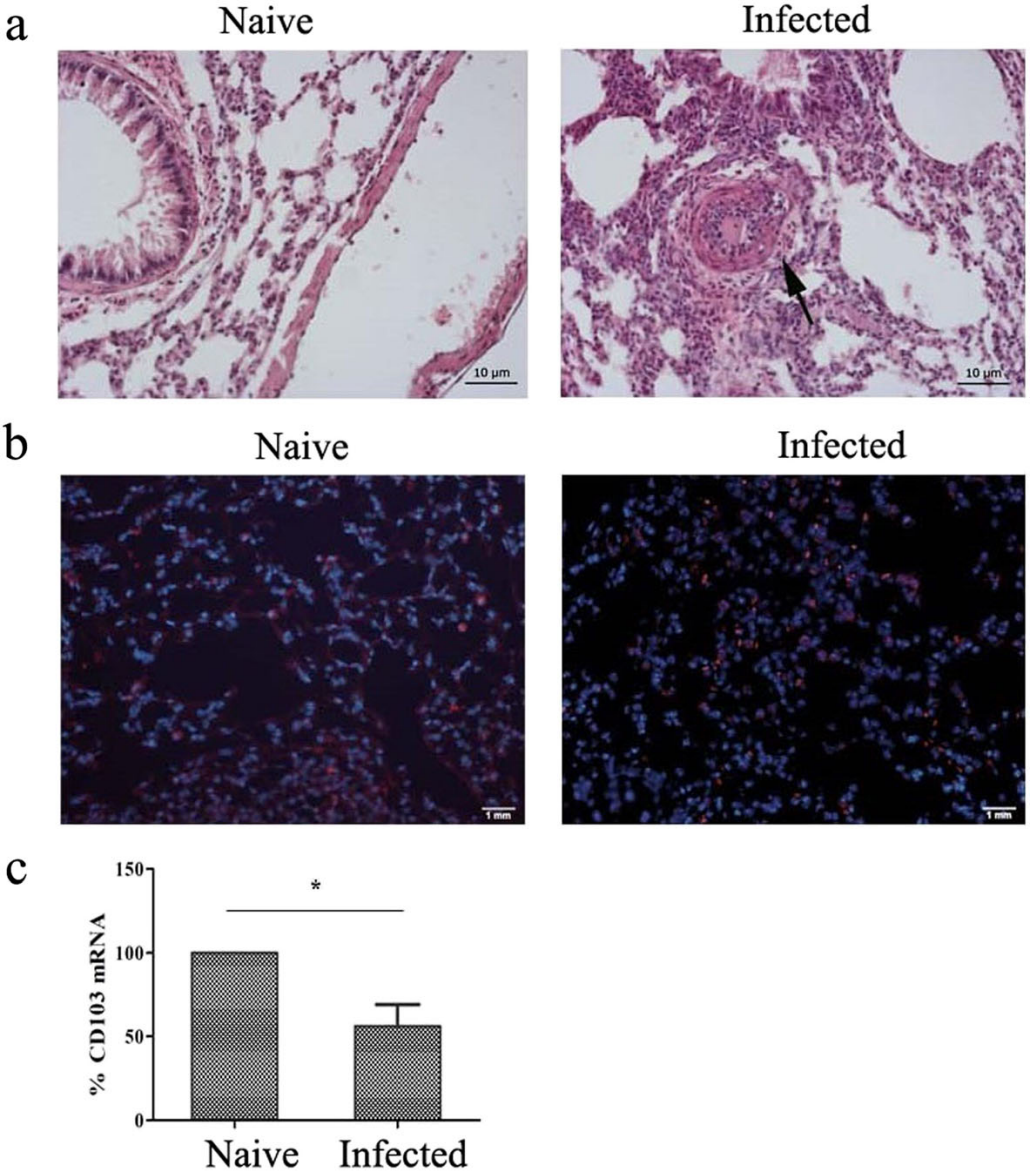
Pathological changes and CD103 expression change in the lungs of infected mice, and distribution of CD103^+^ T cells in infected mouse lung tissue. Mice were percutaneous infected by *S. japonicum*. 5–6 week later, lungs were picked out. (**a**) Hematoxylin and eosin staining was performed on paraffin sections of lung tissue of infected mice. Observing the pathological changes of lung tissue at 20 times mirror, black arrows showing a egg granuloma. Lung tissue sections from naive mice were used as control. (**b**) Fluorescence staining was performed on frozen sections of lung tissue of naive and infected mice. CD103 is indicated by red fluorescence (Alexa Fluor 647, Red), whereas nuclei are visualized using DAPI staining (Blue). A representative result of three independent results is shown. (**c**) Total RNA of lung tissues from both naive and infected mice were extracted, cDNA was synthesized, and fluorescence quantitative PCR was performed to identify the expression of CD103 gene. Results expressed as percentage of gene expression to the same genotype. Data was from three independent experiments with 5 mice per group and shown as mean ± SEM. Variable of the data was analyzed by Tukey’s HSD. * for *P* < 0.05

Mice were sacrificed and the lungs were removed 5–6 weeks after infection. Paraffin sections were made, and H&E staining was performed as described in Methods. Obvious lesions and egg granuloma (marked by black arrows) were found in the lung tissues of infected mice (Fig. 1a). Moreover, frozen sections were stained with fluorescence labeled anti-CD103 (Red) and DAPI (Blue), as described in methods. As shown in Fig. 1b, immunofluorescence results indicated that CD103^+^ cells could be detected in both naive and infected mice lungs. The CD103 expressing cells (red) could be detected in the section of lung tissues from both naive and *S. japonicum* infected mice. Furthermore, fluorescence quantitative PCR was performed to identify the expression of CD103 gene. The results showed that the expression of CD103 gene in the lung tissues from infected mice decreased significantly, which was 56.18 ± 12.56% lower than the naive mice (*P* < 0.05, Fig. 1c).

### Detection of CD103^+^ T cells in the lungs of *S. japonicum*-infected mice

**Fig. 2:**
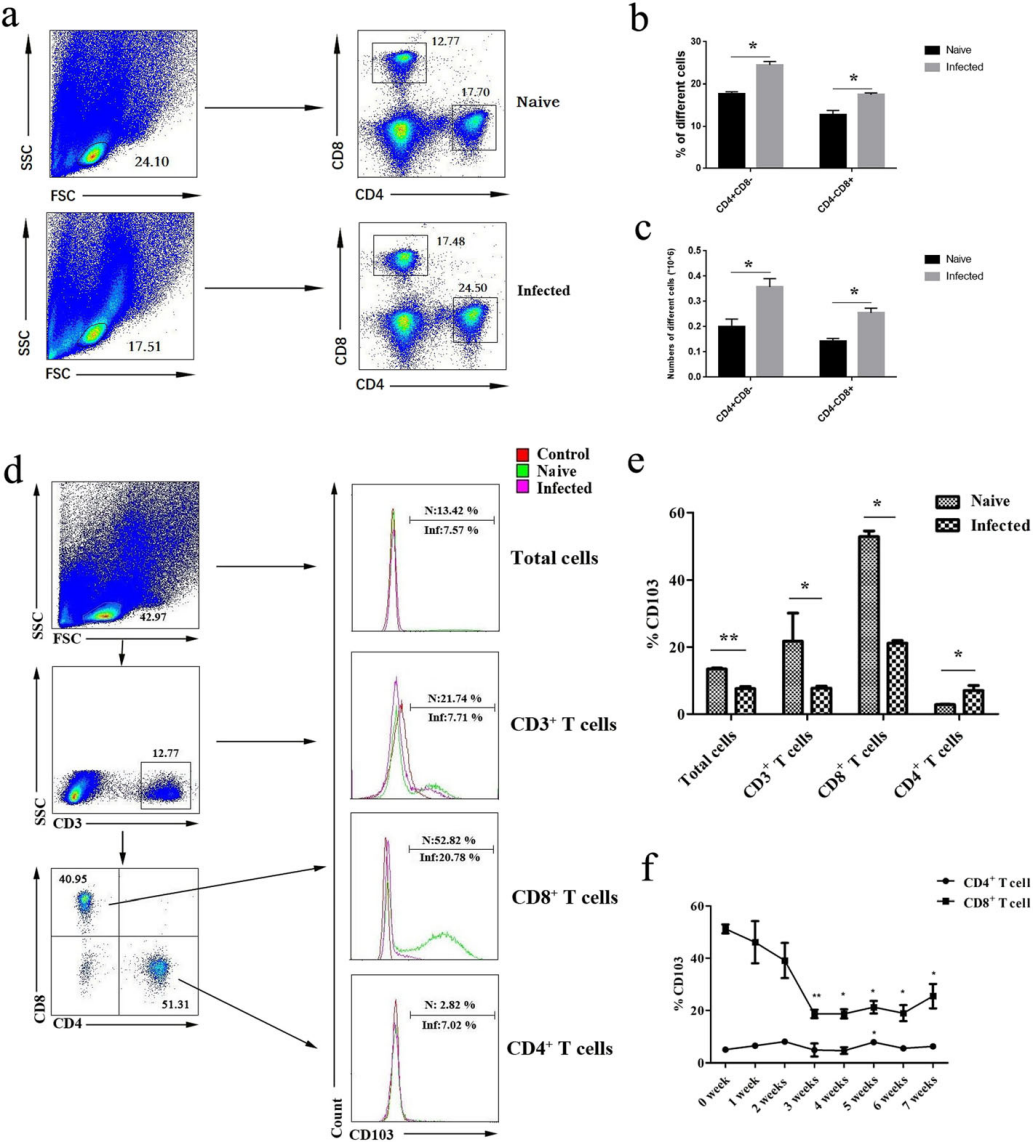
Expression of CD103 on pulmonary CD4^+^ T cells and CD8^+^ T cells. Mice were percutaneous infected by *S. japonicum*. 5–6 week later, lungs were picked out. Single cell suspensions of lungs were isolated from naive and infected mice. (**a**) The percentage of CD4^+^ and CD8^+^ T cells in the isolated lymphocytes were detected by FACS. (**b**,**c**) The percentage and absolute number of CD4^+^ and CD8^+^ T cells were compared between naive anf infected mice. The expression of CD103 was detected using cell surface staining. The expression of CD103 on CD3^+^, CD4^+^, and CD8^+^ T cells was analyzed using FCM, respectively. (**d**) A representative result was shown. (**e**) Average expression of CD103 was calculated from FACS data. (f) To dynamical observe the expression of CD103 in pulmonary T cells, mouse was sacrificed fron week 1 to week 7 after percutaneous infected by *S. japonicum.* Single cell suspensions of lungs were isolated from naive and infected mice. The average expression level of CD103 on CD4^+^ and CD8^+^ T cells were calculated from FACS data. 5 mice per group and results were shown as mean ± SEM. Variable of the data was analyzed by Tukey’s HSD.* for *P* < 0.05

To detect the content of CD103^+^ T cells in the lungs of *S. japonicum*-infected mice, 5–6 weeks after infection, mononuclear cells were isolated from lungs of both naive and infected mice. Cells were stained, the percentage of CD4^+^ and CD8^+^ T cells in the isolated lymphocytes from naive and infected mouse were compared by FACS (Fig. 2a). As show in Fig. 2b, the percentage of CD4^+^ and CD8^+^ T cells were 24.5 ± 0.71% and 17.5 ± 0.54% in the infected mouse, which were higher than that from the naive mouse (17.7 ± 0.48% and 12.8 ± 1.02%), respectively. In the same time the number of CD4^+^ and CD8^+^ T cells in the naive and infected mouse were counted, and compared. As shown in Fig. 2c, the number of CD4^+^ and CD8^+^ T cells in the infected mouse were 3.57 ± 0.37 × 10^5^ and 2.55 ± 0.22 × 0^5^, which were higher than that from the naive mouse (2.0 ± 0.37 × 10^5^ and 1.43 ± 0.12 × 10^5^), respectively.

Moreover, the expression of CD103 was detected on CD3^+^ T cells, CD4^+^ T cells, and CD8^+^ T cells by FACS as shown in Fig. 2d/e. Results showed that the percentage of CD103^+^ cells was 7.57 ± 0.67% in infected mice, which was lower than that of naive mice (13.42 ± 0.32%, *P* < 0.05). The percentage of CD3^+^CD103^+^ T cell in infected mouse lungs was also lower than in naive mice (7.71 ± 0.62% VS 21.74 ± 8.41%, respectively, *P* < 0.05). Further analysis showed that the expression of CD8^+^CD103^+^ T cells (21.12 ± 0.79%) in infected mice was significant decreased when compared with naive mice (52.82 ± 1.75%, *P* < 0.05). In contrast, the expression of CD4^+^CD103^+^ T cells in infected mice (7.02 ± 1.47%) was obviously increased when compared with naive mice (2.82 ± 0.17%, *P* < 0.05). Moreover, levels of CD103 in T lymphocytes were weekly detected in the lung tissues of both naive mice and *S. japonicum*-infected mice for seven weeks after infection. As shown in Fig. 2f, at the first week and second week after infection, the percentage of CD103 expressing cells in CD8^+^ T was slightly reduced (*P* > 0.05). The expression of CD103 in CD8^+^ T cells achieved the lowest level at the third week after infection (*P* < 0.05), and then it kept at the low level without significant changes from week 3 to week 6 (*P* < 0.05). On week 7, the expression of CD103 increased slightly, compared to week 6 (*P* < 0.05). In the eighth week after infection, all the mice were severely sick and were sacrificed for humanity. The change of CD103 expressing cells in CD4^+^ cells was not so obviously. As shown in Fig. 2f, obvious increase of CD103^+^ CD4^+^ T cells could be detected only in week 5 after infection (*P* < 0.05).

### The expression of CD69, CD62L and CD107a on CD103^+^ T cells

**Fig. 3:**
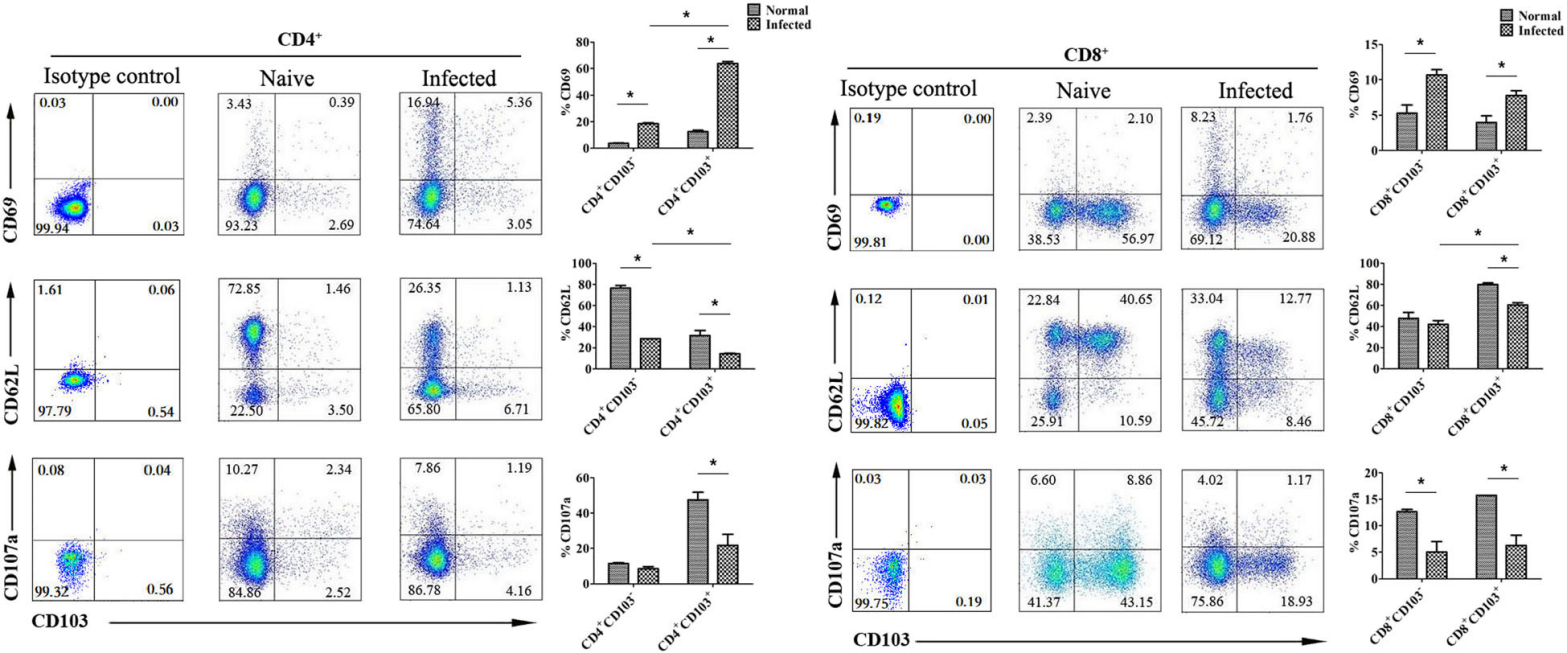
The expression of CD69, CD62L and CD107a on CD103 expressing CD4^+^ and CD8^+^ T cells. Mice were percutaneous infected by *S. japonicum*. 5–6 week later, lungs were picked out. Single lung cell suspensions were separated from naive and infected mice, and stained with monoclonal antibodies against mouse CD4, CD8, CD103, CD69, CD62L and CD107a. The average expression level of CD69, CD62L and CD107a on CD4^+^ and CD8^+^ CD103^+^ T cells were detected by the means of cell surface staining, results were calculated from FACS data. Data was from three independent experiments with 5–6 mice per group and shown as mean ± SEM. Variable of the data was analyzed by the Tukey’s HSD. * for *P* < 0.05

To determine the state of CD103^+^ T cells in the lungs of *S. japonicum* infected mice, single lymphocyte suspensions were isolated from naive and *S. japonicum*-infected mice lungs, and stained. The levels of CD69, CD62L and CD107a were detected on pulmonary CD103-expressing CD4^+^ and CD8^+^ T cells by FACS, respectively. As shown in Fig. 3, the expression of CD69 on CD4^+^CD103^+^ cells in infected mice lungs (63.80 ± 1.49%) was obviously higher than in CD4^+^CD103^+^ cells of naive mice lungs (12.50 ± 1.10%, *P* < 0.05), and CD4^+^CD103^−^ cells of infected mice lungs (18.5 ± 0.75%, *P* < 0.05). On CD8^+^CD103^+^ cells, the percentage of CD69^+^ cells in infected mice (7.79 ± 0.71%) was higher than naive mice (3.97 ± 0.97%, *P* < 0.05). However, it was slight lower than that on CD8^+^CD103^−^ cells (9.84 ± 0.83%, *P* > 0.05). There is an opposite trend toward CD62L and CD107a expression. The expression of CD62L and CD107a on CD4^+^CD103^+^ T cells in infected mice lungs were lower than that in naive mice (CD62L: 14.41 ± 0.52% VS 31.50 ± 4.84%, *P* < 0.05, CD107a: 21.67 ± 6.24% VS 47.57 ± 4.26%, *P* < 0.05). On CD8^+^CD103^+^ cells, the expression of CD62L and CD107a in infected mice lungs was lower than in naive mice lungs (CD62L: 60.38 ± 2.27% VS 79.63 ± 1.66%, *P* < 0.05, CD107a: 6.25 ± 1.96% VS 15.73 ± 0.02%, *P* < 0.05).

### The expression of different cytokines by CD103^+^ T cells

**Fig. 4:**
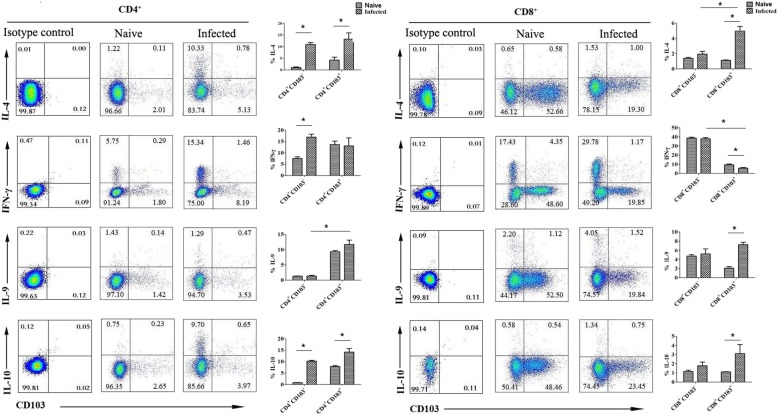
Cytokines produced by CD103 expressing CD4^+^ and CD8^+^ T cells. Mice were percutaneous infected by *S. japonicum*. 5–6 week later, lungs were picked out. Single pulmonary cell suspensions were separated from naive and infected mice, and stimulated with PMA plus ionomycin, the expression of IL-4, IFN-γ, IL-9 and IL-10 were detected by intracellular cytokine staining as described in materials and methods. The average expression of IL-4, IFN-γ, IL-9 and IL-10 on CD4^+^ and CD8^+^ CD103^+^ T cells were calculated from FACS data, respectively. Data was from three independent experiments with 5–6 mice per group and shown as mean ± SEM. Variable of the data was analyzed by the Tukey’s HSD. * for *P* < 0.05

The isolated pulmonary lymphocytes were stimulated with PMA and ionomycin, followed by intracellular cytokine staining and flow cytometry as described in Materials and Methods. CD4^+^ T cells and CD8^+^ T cells were gated first. As shown in Fig. 4, the expression of IL-4, IFN-γ, IL-9 and IL-10 was detected in CD103^+^ T cells. The expression of IL-4 and IL-10 on CD4^+^CD103^+^ T cells in infected mice lungs was significantly higher than in naive mice lungs (IL-4: 13.28 ± 2.66%% VS 4.26 ± 1.25, *P* < 0.05, IL-10: 14.16 ± 1.6% VS 7.86 ± 0.42%, *P* < 0.05), but there was no significant difference in the expression of IFN-γ and IL-9 between the naive and *S. japonicum*-infected CD4^+^CD103^+^ T cells. The percentage of IFN-γ-expressing CD8^+^CD103^+^ T cells in infected mice was 5.73 ± 0.69%, which was lower than in naive mice (9.34 ± 0.89%, *P* < 0.05), and the percentage of IL-4 and IL-9-expressing CD8^+^CD103^+^ T cells in infected mice was 4.96 ± 0.60% and 7.23 ± 0.54%, which was higher than in naive mice (1.11 ± 0.06% and 2.08 ± 0.34%, *P* < 0.05), CD8^+^CD103^+^ T cells. However, there was no difference in the expression of IL-10 between the CD8^+^CD103^+^ T cells in the lungs of naive and *S. japonicum*-infected mice (*P* > 0.05).

## Discussion

In the course of *S. japonicum*-infection, *schistosomulum* might migrate and develop in the lung at the early stage, and eggs laid out by adult worms could deposit in the lung of infected mice at the later stage. These activities would cause acute pulmonary schistosomiasis (APS) [37]. Many kinds of immune cells were involved in the process of this change about 5–8 weeks post infection [12]. In present study, classic egg granulomatous inflammation was found in the lung tissues of *S. japonicum*-infected C56BL/6 mice (Fig. 1a). It suggested the success in construction of an APS model. CD103 molecules were expressed in the tissues of both naive and infected mice (Fig. 1b), the level of its transcript factor was decreased, obviously (*P* < 0.05, Fig. 1c). It means that many CD103 expression cells residue in the lungs of naive mouse, and these CD103 expressed cells involved in the course of *S. japonicum*-infection induced pulmonary inflammatory disease, as found in asthma [38].

It was reported recently that CD103 was an important marker for TRM cells [19]. As we know, T cells, especially CD4^+^ Th cells play important roles in the process of Schistosomiasis [39]. The expression of CD103 molecules was detected on the surface of T cells. Results showed that both CD4^+^ and CD8^+^ T cells could express CD103, and the percentage of CD103^+^ cells in CD8^+^ cells was higher than CD4^+^ cells in pulmonary T cells isolated from naive C57B/6 mice (*P* < 0.05, Fig. 2e), as found in the genital tract of HIV-positive women [40]. It implied that a larger population of CD8^+^ T cells located in the lung. It was reported that increased numbers of (CD103^+^CD8^+^ T cells were found in the airways of smokers with and without chronic obstructive pulmonary disease [41]. The percentage of CD103^+^CD8^+^ cells significantly decreased in this model, especially from week 3 to week5 (*P* < 0.05, Fig. 2e, f). It might relate to the newly recruited CD8^+^CD103^−^ T cells. The number of the CD8^+^CD103^+^ cells was constant in the lung of infected mice 5–6 weeks after infection, for the increased cell number in the lung (Fig. 2c). Although the percentage of CD103^+^CD4^+^ cells slightly increased, obvious increasing was only detected on week 5 after infection (*P* < 0.05, Fig. 2e, f). The percentage and absolute numbers of CD4^+^ cells T cells increase obviously (*P* < 0.05, Fig. 2a-c). It was related to the Th2 dominant immune response induced by the migration of infected *S. japonicum* and its eggs*,* which closely contact with pulmonary immune system [42]. It suggested that CD103^+^CD4^+^ might exhibit more important roles in mediating the immune response induced by *S. japonicum* infection.

CD69 is a membrane-bound, type II C-lectin receptor. It is a classical early marker of lymphocyte activation due to its rapid appearance on the surface of the plasma membrane after stimulation [43]. Recently, CD69 is regarded as a marker of TRM [44]. Like CCR7 and l-selectin, CD62L is a LN homing marker expressed on the surface of both naive and central memory T cells [45]. As shown in Fig. 3, increased CD69, decreased CD62L expressions were detected on both CD4^+^ and CD8^+^ CD103 expressing T cells in the lungs of infected mice (*P* < 0.05). CD4^+^ CD103^+^ T cells from infected mice expressed significantly higher level of CD69, and lower level CD62L molecule than CD8^+^ CD103^+^ T cells (*P* < 0.05). It indicated that both CD103-expressing CD4^+^ and CD8^+^ T cells were involved in the infection induced pulmonary inflammation, and CD4^+^ Th cells might exhibit more function in this process. Moreover, the expression of cytotoxicity associated molecule CD107a was detected in CD103-expressing T cells [46]. As shown in Fig. 3, CD107a was found to be decreased in both CD4^+^ and CD8^+^ CD103^+^ T cells in the lungs of *S. japonicum*-infected mice (*P* < 0.05). It suggested that the cytotoxicity role of pulmonary CD103^+^ cell population is limited in the course of *S. japonicum* infection.

IFN-γ and IL-4 were classic cytokines secreted by Th1 and Th2 cells, respectively. Especially, IL-4 was the main cytokine mediating the immune response against *S. japonicum* [11]. As shown in Fig. 4, significantly higher percentages of IL-4^+^ cells were found in both CD4^+^ and CD8^+^ CD103-expressing pulmonary T cells from infected mice (*P* < 0.05). Similar with our study, high levels of Th2 cytokine secreting resident memory CD4^+^ T cells were found in intestinal helminth infected mice [47]. Moreover, the percentage of IL-4^+^ cells in CD103^+^CD8^+^ T cells was obviously higher than CD103^−^CD8^+^ T cells, and the percentage of IFN-γ^+^ cells in CD103^+^CD8^+^ T cells was significantly lower than CD103^−^CD8^+^ T cells (*P* < 0.05). It indicated that CD103 expressing TRM cells might be a source of IL-4-secreting CD8^+^ Tc2 cells in the course of *S. japonicum* infection as reported by Xu et al [48]. It suggested that TRM cells will enhance *S. japonicum* infection induced granuloma inflammation in the lung.

Mainly secreted by Th9 cells, IL-9 could stimulate mast cell accumulation in tissues, promote lymphocytes survival, enhance class switching to IgE in B cells, and alter hematopoietic progenitor cell activity, for its receptor expressed on multiple cell types [49]. Recently, IL-9 was reported to be secreted by Tc cell in many diseases [50, 51]. IL-9 was found to be able to mediate *S. japonicum* infection induced hepatic damage in our previous study [6]. However, it is interesting to find that in *S. japonicum*-infected mouse lung, the percentage of IL-9^+^ cells increased significantly only in CD103-expressing CD8^+^ T cells (*P* < 0.05, Fig. 4). It indicated that CD103^+^CD8^+^ TRM cells might be the main source of *S. japonicum* infection induced IL-9 in the lung. IL-10 was regulatory cytokine, which could be secreted by CD4^+^Foxp3^+^ Treg cells, Tr1, Th3, and Treg [20]. As shown in Fig. 4, significant higher percentage of IL-10-producing CD103^+^CD4^+^ cells was found in the lung of *S. japonicum*-infected mouse. It suggested the existence of regulatory function in the CD4^+^CD103^+^ TRM cells.

## Conclusions

In this manuscript, obvious changes in the content, memory related molecules expression, and cytokines producing were found in pulmonary CD103 expressing CD4^+^ and CD8^+^ T cells from *S. japonicum* infected mice. It demonstrated that CD103-expressing pulmonary CD4^+^ and CD8^+^ T cells played important roles in mediating *S. japonicum* infection induced granulomatous inflammation in the lung.

## Data Availability

The data used in the current study are available from the corresponding author on reasonable request.

## Abbreviations

TRM: Tissue-resident memory T cells
*S. japonicum*: *Schistosoma japonicum*
Q-PCR: Quantitative PCR
FCM: Flow cytometry
HBSS: Hank’s balanced salt solution
FBS: Fetal bovine serum
H&E: Hematoxylin-eosin
PBS: Phosphate buffered saline
PMA: Phorbol 12-myristate 13-acetate
BFA: Brefeldin A
